# Polaronic Mass Enhancement and Polaronic Excitons in Metal Halide Perovskites

**DOI:** 10.1021/acsenergylett.4c00905

**Published:** 2024-05-13

**Authors:** Michal Baranowski, Andrzej Nowok, Krzysztof Galkowski, Mateusz Dyksik, Alessandro Surrente, Duncan Maude, Marios Zacharias, George Volonakis, Samuel D. Stranks, Jacky Even, Miroslaw Maczka, Robin Nicholas, Paulina Plochocka

**Affiliations:** † Department of Experimental Physics, Faculty of Fundamental Problems of Technology, Wroclaw University of Science and Technology, 50-370 Wroclaw, Poland; § Laboratoire National des Champs Magnétiques Intenses, EMFL, CNRS UPR 3228, Université Toulouse, Université Toulouse 3, INSA-T, 31400 Toulouse, France; ∥ Université Rennes, INSA Rennes, CNRS, Institut FOTON - UMR 6082, F-35000 Rennes, France; ⊥ Université Rennes, ENSCR, INSA Rennes, CNRS, ISCR - UMR 6226, F-35000 Rennes, France; # Cavendish Laboratory, University of Cambridge, JJ Thomson Avenue, Cambridge CB3 0HE, United Kingdom; ∇ Department of Chemical Engineering and Biotechnology, University of Cambridge, Philippa Fawcett Drive, Cambridge CB3 0AS, United Kingdom; ○ Institute of Low Temperature and Structure Research, Polish Academy of Sciences, ul. Okolna 2, 50-422 Wroclaw, Poland; ⧫ Department of Physics, Clarendon Laboratory, University of Oxford, Parks Road, Oxford OX1 3PU, United Kingdom

## Abstract

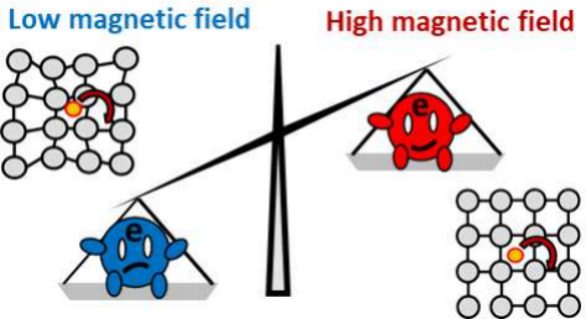

In metal halide perovskites, the complex dielectric screening together with low energy of phonon modes leads to non-negligible Fröhlich coupling. While this feature of perovskites has already been used to explain some of the puzzling aspects of carrier transport in these materials, the possible impact of polaronic effects on the optical response, especially excitonic properties, is much less explored. Here, with the use of magneto-optical spectroscopy, we revealed the non-hydrogenic character of the excitons in metal halide perovskites, resulting from the pronounced Fröhlich coupling. Our results can be well described by the polaronic-exciton picture where electron and hole interactions are no longer described by a Coulomb potential. Furthermore, we show experimental evidence that the carrier-phonon interaction leads to the enhancement of the carrier’s effective mass. Notably, our measurements reveal a pronounced temperature dependence of the carrier’s effective mass, which we attribute to a band structure renormalization induced by the population of low-energy phonon modes. This interpretation finds support in our first-principles calculations.

Interaction between charge carriers and lattice excitation (phonons) has a profound impact on the optical response of semiconductor materials, determining transition line width or fulfilling energy-momentum conservation requirements in indirect light absorption and emission processes.^1,2^ The coupling of the carriers to optical lattice vibrations can also significantly affect the spectrum of the excitonic transitions,^3−6^ leading to a deviation from the hydrogenic exciton model^2^ (commonly used for many semiconductors) or enhancing carrier mass.^7,8^ This is especially important for ionic semiconductors, where carrier-optical phonon coupling is strong. For a long time, ionic semiconductors have stayed in the shade of covalent semiconductors (III–V, II–VI), where the impact of the phonons on the exciton energy structure could be neglected (the hydrogen model works very well). However, the recent boom in the field of metal-halide perovskites has brought the discussion of the impact of polaronic effects on the excitonic properties into the spotlight of scientific interest.^4,8−14^

Tremendous interest in metal-halide perovskites has been triggered by skyrocketing progress in energy harvesting application,^15−18^ followed by promising results in the field of light emitters and detectors.^19,20^ At the same time, perovskite semiconductors constitute a fascinating and challenging material system for fundamental understanding as they bridge inorganic and organic semiconductors.^10^ Despite extensive research on the physics of these materials, a complete and unified understanding of their electronic and optical properties is still missing.^8−11^ The crucial features of perovskites, which distinguish them from regular inorganic semiconductors (GaAs, Si, GaN), are the richness of their lattice dynamics^21−25^ and the related complex dielectric response.^23,26^ Indeed, the soft ionic and anharmonic lattice makes perovskites an intriguing system for the investigation of carrier–lattice coupling.^8,10,27^ The coupling of carriers to anharmonic vibrations plays a key role around room temperature and above and is essential to explaining the mild bandgap change across-phase transitions,^27−31^ the temperature dependence of carriers mobility,^10,32^ or thermal conductivity.^30^ At very low temperatures, anharmonicity-related effects are expected to be weak; however, the carrier-phonon coupling still significantly influences the optoelectronic properties of these materials.

In metal halide perovskites, the non-negligible coupling of carriers with optical phonons and formation of polarons is expected due to significant contrast between high ε_∞_ and low ε_s_ frequency parts of the dielectric function^12,25,26^ and the low energy of some phonon modes.^25^ This interaction quantified by the Fröhlich coupling constant (α = 1.7–2.2)^25^ is orders of magnitude larger in perovskites than in GaAs (α = 0.068) or other III–V or II–VI semiconductors.^33^ Therefore, it should have a profound impact on the electronic and optical properties of perovskites and cannot be ignored as is often the case with other semiconductor materials. For example, Fröhlich coupling is known to enhance the effective masses,^7,34^m* ≈ *m*(1 + α/6), which is one of the most crucial parameters for device operation and should therefore be thoroughly understood.

The concept of large polarons (i.e., carriers dressed with the lattice interaction) has already been used to explain some of the puzzling aspects of carrier transport in perovskites.^8,10,25,35−40^ Yet, the possible impact of polaronic effects was mostly neglected in the analysis of the optical response, leading to confusing reports.^4,9^ For instance, the excited excitonic states have been hardly observed even for the highest quality crystals,^41−43^ clearly deviating from the hydrogen-like exciton model^4^ (where the oscillator strength of the 2s states is 1/8 of the 1s state). More importantly, there are evident discrepancies in the reported carrier mass values determined through magneto-optical studies performed in the low^41,44^ and the high field limits.^9,45,46^ The higher effective mass is consistently observed in the former case, providing another indication of the importance of carrier–phonon coupling in perovskites for their optical response.

Here, we employ magneto-optical spectroscopy and exciton-polaron modeling to show that the above discrepancy can be reconciled by taking into account the impact of the Fröhlich coupling on the optical response of metal halide perovskites. By solving the exciton-polaron model^3−6^ in the magnetic field, we prove that the contrasting values of the effective masses observed in the low and high magnetic field, regime stem from the polaronic nature of excitonic transitions. In this way, we provide direct evidence of the importance of Fröhlich coupling for understanding the optical response and carriers’ effective mass in perovskite crystals. Moreover, we highlight the importance of carrier–phonon coupling for band structure renormalization, which leads to strong temperature dependence of the carrier’s effective mass.

We perform our investigation for three high-quality perovskite single crystals: MAPbI_3_, MAPbBr_3_, and CsPbBr_3_ (for synthesis details, see the Methods section). To reveal the impact of polaronic effects on carriers’ mass, we measured the optical response in a pulsed magnetic field up to 90 T. In the low field limit, when ℏω_c_ < ℏω_LO_ (where ω_c_ = *eB*/μ is a cyclotron frequency, ℏω_LO_ is the LO phonon energy, and μ is a reduced carrier mass probed by interband transitions 1/μ = 1/*m*_*h*_ + 1/*m*_*e*_), the optical properties are determined by the polaron mass as the lattice follows the cyclotron motion of the carriers. In the high field limit when ℏω_c_ ≫ ℏω_LO_, the bare carrier mass μ is probed because lattice deformation cannot follow fast cyclotron movement of the carrier.^47^ Assuming reduced carrier mass μ in the range of 0.1–0.15*m*_0_^45^ (where *m*_0_ is free electron mass) and an effective LO phonon energy ∼ 15 meV,^25,48^ the crossover between the two magnetic field regimes should occur around ∼20 T. Therefore with the available magnetic field range, we can probe optical response related to the polaron mass and bare carrier mass limit.^47^

Figure 1a shows the reflection spectrum of MAPbBr_3_ measured at 2 K (blue curve) together with its derivative (yellow curve), whose minima correspond to the energy of the excitonic transitions^42^ (for other samples, see Figures S1 and S2). The optical response is dominated by the strong 1s excitonic transition at 2.249 eV, which, on the high energy side (2.262 eV), is followed by a much weaker 2s exciton state. The 2s transition oscillator strength is much lower than 1/8 of the 1s strength (∼2–3% instead of ∼12%) which is a first hallmark of the polaronic character of the observed excitonic transition.^4^

**Figure 1:**
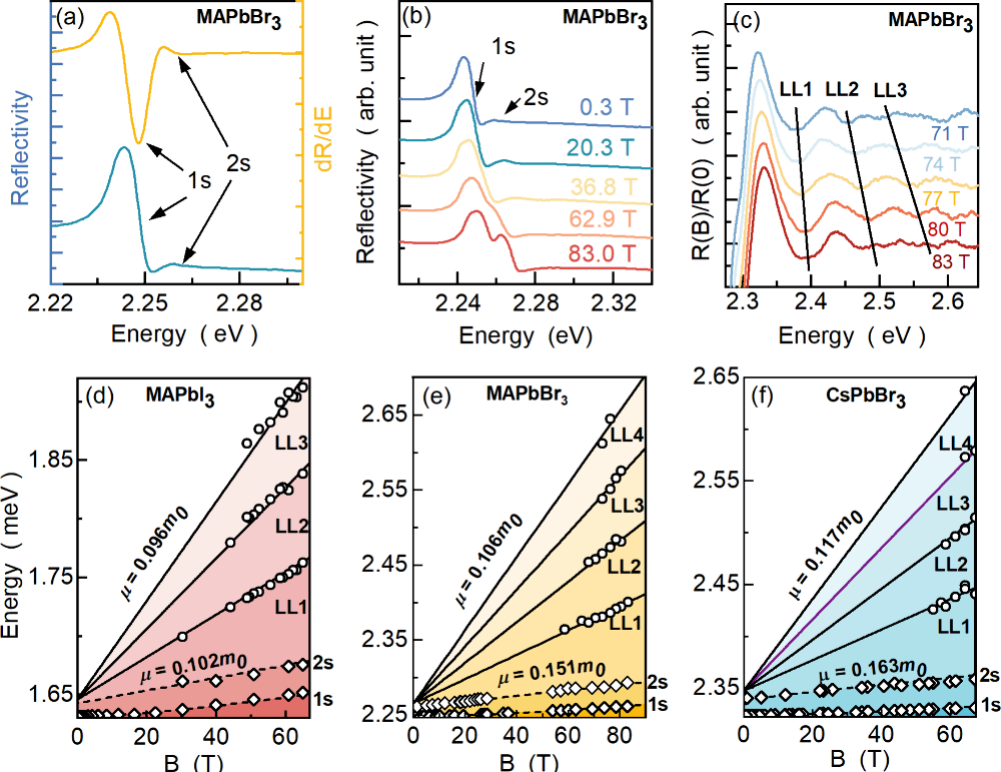
(a) MAPbBr_3_ reflectance spectrum (blue) taken at 2 K and its derivative (yellow) showing resonance features related to 1s and 2s excitonic transitions. (b) Evolution of reflectance spectrum in magnetic field. (c) Rationed spectra of reflectance for different magnetic field values; black lines indicated Landau level (LL) transitions. (d–f) Fan charts summarizing interband Landau level transition energies as a function of the magnetic field for MAPbI_3_, MAPbBr_3_, and CsPbBr_3_. Efectice reduced mass μ with the use of eq 1.

To probe the reduced effective mass, we investigated exciton and Landau level transitions evolution in the presence of high magnetic fields. The evolution of the reflection spectrum of MAPbBr_3_ is presented in Figure 1b (for remaining crystals, see the SI). Both excitonic transitions blue-shift, and at higher magnetic fields Zeeman splitting of the excitonic states can be observed. To reveal the transitions between interband Landau levels in Figure 1c, we plot spectra obtained at a nonzero magnetic field divided by the spectrum measured at zero magnetic field. Such ratioed spectra exhibit equally spaced features (on the high energy part of the spectrum), which we ascribe to the interband Landau levels transitions. The evolution of these interban Landau level transitions is summarized in panels d–f of Figure 1. This dependence can be well fitted with a standard formula for parabolic band dispersion (since the excitonic corrections to the higher Landau levels are very small):^45,47,49^
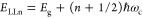
1which provides a very direct measure of carriers’ reduced mass μ, which are 0.096, 0.106, and 0.117 masses of electrons for MAPbI_3_, MAPbBr_3_, and CsPbBr_3_ respectively (see also Table 1). It is important to note that the Landau levels could be observed only in the field above 40–50 T; therefore, we can safely assume that the extracted values correspond to the masses of bare carriers.

**Table 1:** Summary of the Extracted Parameters^a^

	μ	μ_*x*_	μ*	ε_∞_	ε_*s*_	*E*_*LO*_	α
MAPbI_3_	0.096	0.102	0.114	7	15	12 meV	1.12
MAPbBr_3_	0.106	0.151	0.137	5.2	16	15 meV	1.75
CsPbBr_3_	0.117	0.163	0.158	4.5	16	18 meV	2.1

a μ, reduced effective mass determined based on Landau level transitions; μ_X_, effective mass extracted from hydrogenic exciton model; μ*, polaron mass calculated according to eq 5 and eq 6 with parameters ε_∞_, ε_s_, and *E*_LO_ obtained through modelling of excitonic transition with the use of Bajaj potential.

To measure the effective reduced mass at the low magnetic field limit, we make use of excitonic transitions which can be observed regardless of field strength. Thanks to the superior quality of our crystals, both 1s and 2s excitonic transions can be observed. Their evolution in the magnetic field (for all three crystals) is summarized in Figure 2 as diamond points (the contribution of Zeeman splitting is canceled out by averaging the energy of split states). At first, we model the behavior of 1s and 2s exciton states in a magnetic field using the numerical solutions of the hydrogen atom in a magnetic field proposed by Makado and McGill.^50^ In this approach, the energy shift induced by the magnetic field depends (only) on the dimensionless parameter γ = ^1^/_2_ℏω_c_/*E*_B_, which involves the exciton binding energy *E*_B_ and the reduced effective mass used for the cyclotron energy. The simultaneous observation of 1s and 2s exciton states delivers strong constraints for the exciton binding energy; therefore, effectively, μ becomes the only fitting parameter. As is shown by the dashed red lines, magnetic field-induced shifts of the 1s and 2s states as well as their energy distance can be reasonably reproduced with the hydrogenic model. However, significantly higher values of μ (higher than the one extracted from Landau levels observed at high magnetic field values see Table 1) have to be used to fit experimental data well. In other words, the excitons behaved if they were composed of heavier carriers than those probed by Landau level analysis (which is sensitive to the bare carrier mass). This is a smoking gun for the polaronic mass enhancement of the carriers in metal-halide perovskites. The usage of bare carrier masses (in the hydrogen exciton model) fails to fit the data (especially in Br-containing compounds), as shown by short-dashed red lines.

**Figure 2:**
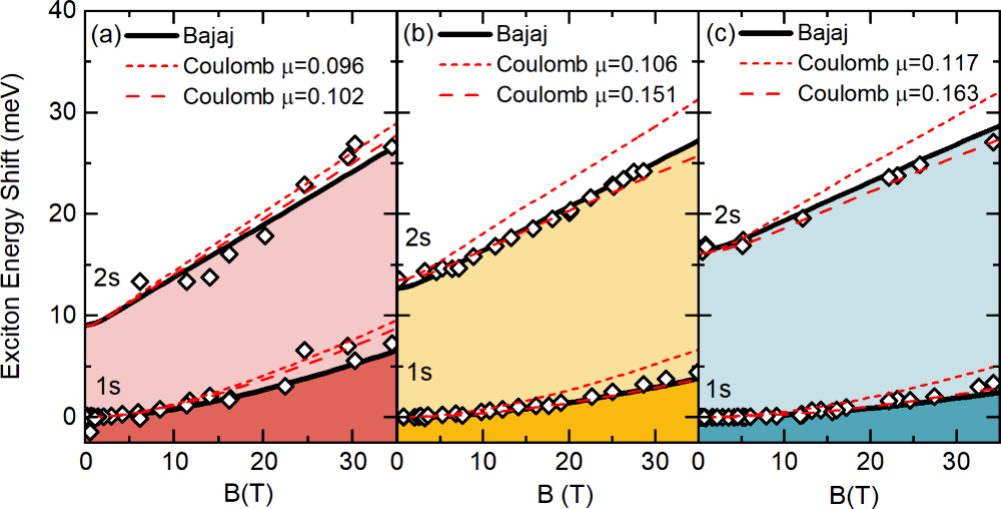
Dependence of 1s and 2s excitonic transition energy as a function of magnetic field (open points) for MAPbI_3_, MAPbBr_3_, and CsPbBr_3_ crystals at 2 K. The dashed red line represents the best fitting with the hydrogen model, while the short-dashed red line shows a prediction of the hydrogen model with the μ extracted from Landau level spectroscopy. The black line shows modeling with the use of Bajaj potential and parameters summarized in Table 1.

At this point, it is important to emphasize that in ionic crystals the hydrogenic exciton model should be treated as a simplified picture of bound electron–hole pairs.^3−6,51^ More precise descriptions should take into account the contrasting low- and high-frequency parts of the dielectric function, which cause the electron–hole interaction to no longer be described by a simple Coulomb potential, and that the carrier’s mass can be enhanced.^3−7^ Therefore, to further support the exciton-polaron character of the observed transition, we use the effective Hamiltonian for excitons in polar semiconductors proposed by Bajaj^6^ to model the energies of exciton states energy in the magnetic field. The Bajaj potential is a phenomenological modification of the Haken potential,^3^ which remains in quantitative agreement with more advanced models^5,51^ (as we show in Table S1) and can be easily extended for the case of a magnetic field (see detailed description in the SI). It has the following form:
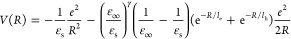
2where *l*_e/h_ is an electron and hole polaron radius defined as
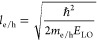
3The extra factor (ε_∞_/ε_s_)^γ^ (which is not present in the Haken potential) provides improved agreement with the experimentally measured values of exciton binding energy. For a range of polar crystals, including the high α value halides, it was shown that γ = 3/5 is the optimal choice.^6^ We solve numerically the Schrödinger equation for the exciton-polaron problem in a magnetic field, which for the optically active s states take the following form (see SI for detailed description):
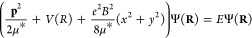
4where **p** is the momentum operator, *e* is the elementary charge, and *x* and *y* are spatial coordinates in the plane perpendicular to the magnetic field direction. The μ* is the reduced polaron mass, 1/μ* = 1/*m*_e_* + 1/*m*_h_*, where
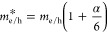
5and α is a Fröhlich coupling constant:
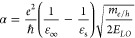
6In the modeling, we used the bare carrier reduced mass μ extracted from the Landau level analysis (assuming the same mass for the electron and the hole) as the input parameter, and polaron mass was calculated based on equations 6 and 5. The results of the simulation are presented as black lines in Figure 2, which shows very good agreement with experimental data for the reasonable values (summarized in Table 1) of ε_∞_ and *E*_LO_.^25,26,48,52,53^ It is important to note that parameters used in the modeling should be treated as effective values because the model used (and all Haken model developments) assumes coupling to only one optical phonon mode. This “effective” character might be the reason for somehow lower values of ε_s_ than the ones typically reported.^25,26^ Nevertheless, our model allows us to very well describe 1s and 2s excitonic transition behavior in metal halide perovskites under the presence of a magnetic field. Therefore, we show that the effective mass probed in the low and high field limits can be reconciled by taking into account the Fröhlich coupling and its impact on the excitonic properties of the material. In contrast to the simple hydrogenic model, our approach relates the clear mass enhancement to the dielectric properties of the material, providing very direct evidence for the polaronic nature of the excitons in metal halide perovskites and the importance of Fröhlich coupling for the optical response of these materials.

The measured Fröhlich coupling scales with the bandgap or halide anion as can be seen from Table 1 and the deviation from the hydrogen model is more evident for Br compounds. This can be understood as an effect of stronger ionic bonding of the metal with the halide, when I is exchanged to Br or Cl, which leads to a lower value for the effective screening.^14,46^ This scaling of the Fröhlich coupling also indicates that the high effective mass of exciton observed in CsPbCl_3_^54^ might partially stem from a stronger polaronic enhancement. At the same time, the impact of the A-site cation is not very pronounced (see Table 1) showing that polaronic properties of perovskites are dominated by LO phonon modes of the inorganic metal-halide sublattice, in agreement with previous works.^14,21^

We have also explored the evolution of the bare carriers’ mass with temperature. Generally, in semiconductors, the temperature dependence of the effective carrier mass is expected due to the variation of the band gap with temperature.^47,55,56^ In perovskites, unusually for semiconductors, the bandgap opens with increasing temperature; therefore, an increase of the effective mass is expected as temperature increases. Indeed this effect can be observed via Landau level spectroscopy, as shown in Figure 3. Evidently, the slope of the Landau level fan chart decreases as temperature rises from 2 to 60 K (see blue and red lines) pointing to an increasing reduced effective mass μ. The same behavior can be observed by analyzing the excitonic transitions as shown in Figure S3. A similar dependence of μ vs *E*_g_ is consistently observed for all investigated compounds, which is summarized in panel b of Figure 3.

**Figure 3:**
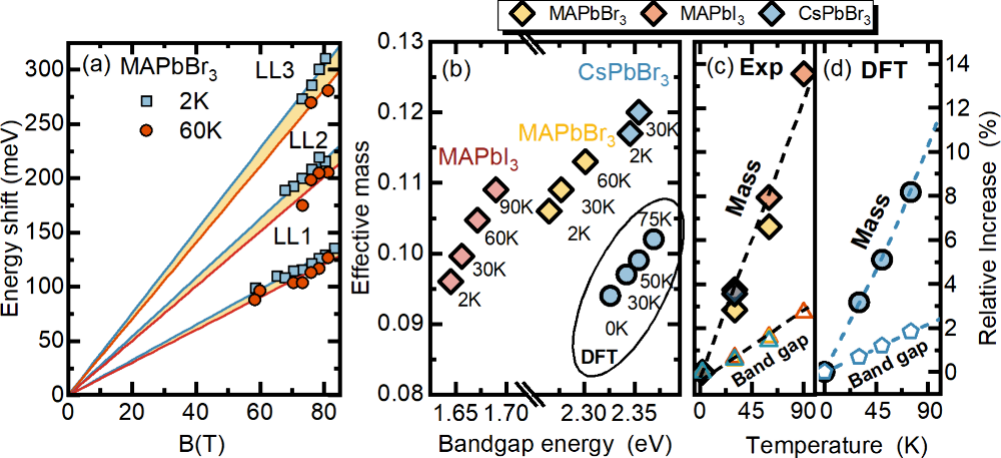
(a) Comparison of the Landau level shifts at 2 K (blue) and 60 K (red) for MAPbBr_3_. The different slope of two fan charts is highlighted by the yellow filling. (b) Summary of the bare reduced effective mass μ for all three compounds at different temperatures. Diamonds correspond to experimental results, while circle points are DFT calculations. (c) Experimentally measured temperature-induced relative increase of the bandgap and μ. (d) Calculated temperature-induced relative increase of the bandgap and μ.

In metal halide perovskites, the relation between the effective mass and the bandgap can be well described with the simple linear dependence^45,49^ (which comes from the two-band **k**·**p** model (see the SI for details):
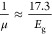
7This simple model successfully predicts the value of effective mass, measured at 2 K for a broad range of metal–halide perovskite compounds characterized by different bandgaps.^49^ However, at the same time, it fails to explain the magnitude of the effective mass increase induced by temperature. It is straightforward to show from eq 7 that the relative increase of mass should be the same as the bandgap: μ(*T*)/μ(2 K) = *E*_g_(T)/*E*_g_(2 K). As shown in Figure 3c, this is not the case, and mass increases much faster. More precise 4-band **kp** with generally accepted band structure parameters^57^ gives the same result as shown in the SI.

This strong temperature dependence is another indication of carrier-phonon interaction in metal halide perovskites, which leads to the renormalization of band dispersion when low-energy phonon modes become populated with increasing temperature. To illustrate this phenomenon, we conducted first-principles calculations on the orthorhombic CsPbBr_3_ band structure, explicitly considering the influence of electron–phonon coupling. This analysis was performed using the special displacement method (SDM)^58,59^ within the harmonic approximation as implemented in the EPW/ZG module.^60^ Special displacements were generated for a 4 × 2 × 2 supercell containing 320 atoms, using the phonons that incorporate the effect of long-range interactions. The calculated phonon band dispersion is shown in Figure S5 in the SI. The impact of spin–orbit coupling was included in all SDM supercell calculations. The effective masses were determined using finite differences and taking the average of the diagonal elements of the effective mass tensor. The calculated values of the bandgap and effective masses as a function of temperature are summarized in Table S2. The corresponding reduced mass μ of carriers is also shown in Figure 3b as a circle point. The calculated values for μ lie within 0.095–0.105 in the temperature range 0–75 K and underestimate the experimental data by around 20% but still follow the experimental trend. However, as shown in panel d of Figure 3, the temperature-induced relative increase of the effective masses and the bandgap show very good agreement with the measured values, illustrating the importance of electron–phonon interaction for temperature evolution of the effective mass. The absolute values from DFT could be adjusted by considering quasiparticle corrections within the GW approximation, which increase the effective mass by ∼25%,^61^ but they are not expected to qualitatively change the whole picture, inducing only small quantitative changes^34^ in the relative increase of the effective masses.

In summary, our study highlights the polaronic nature of charge carriers in metal halide perovskites, revealing the crucial role of Fröhlich coupling in shaping their optical response. The observed differences in the effective carrier mass between low and high magnetic field limits highlight the importance of this interaction for the optical properties of these materials. With the use of the Bajaj exciton-polaron model, accounting for the dielectric properties, we have shown that the contrasting values of the effective mass can be reconciled corroborating the polaronic and non-hydrogenic character of the excitons in perovskites. Moreover, our research uncovers a unique temperature-dependent increase in carriers’ effective mass, which results from the band structure renormalization when the low-energy phonons become populated as shown by first-principles calculations. The highlighted impact of carrier-phonon coupling on the carrier mass enhancement and the optical properties shows that polaronic effects cannot be neglected in the intended optoelectronic applications.

## Methods

Reflection spectra as a function of the magnetic field were measured in a pulsed field magnet with maximum field **B** = 90 T and the whole pulse duration of ∼100 ms. Broad-band white light was provided by a tungsten halogen lamp and directed on the sample inside the magnet with the use of an optical fiber. The magnetic field measurements were performed in the Faraday configuration with the light propagating parallel to the magnetic field. The reflected signal was collected by a fiber bundle, which surrounds the excitation fiber (backscattering geometry). The signal was spectrally analyzed with the use of a grating monochromator and equipped with a liquid-nitrogen-cooled CCD camera. The sample was placed in a liquid helium cryostat inside the magnet.

Single crystals of all perovskites were grown using the antisolvent vapor-assisted crystallization method, in which the appropriate antisolvent is slowly diffused into a solution containing the crystal precursors. In the case of MAPbBr_3_, 1 mL of 2 M solution of methylamine in methanol (2 mmol, Sigma-Aldrich) and 2 mmol of HBr (48 wt % in H_2_O, Sigma-Aldrich) was mixed with 5 mL of acetonitrile (Sigma-Aldrich). Then, 2 mmol of PbBr 2 (98%, Sigma-Aldrich) was added to the above-prepared amine hydrobromide solution under stirring. In the next step, dimethylsulfoxide (DMSO, Sigma-Aldrich) was added drop by drop until the orange precipitate, which appeared in the solution, disappeared completely. The clear solution was transferred into a glass vial, and this vial was placed in a second larger glass vial containing methyl acetate (99.5%, Sigma-Aldrich). The lid of the outer vial was thoroughly sealed, but the lid of the inner vial was loosened to allow diffusion of the methyl acetate into the precursor solution. Orange crystals, which grew at the bottom of the vial, were separated from the mother liquid and dried at room temperature. A precursor of MAPbI_3_ was prepared by dissolving 2 mmol of PbI_2_ (99%, Sigma-Aldrich) in 0.5 mL of HI (57 wt % in H_2_O, stabilized with H_3_PO_2_, Sigma-Aldrich). Then, 2 mmol of methylamine was added, leading to the precipitation of a black powder. In the next step, acetonitrile was added drop by drop under constant stirring until the solution became clear. This solution was transferred into a glass vial, and this vial was placed in a second larger glass vial containing methyl acetate. The grown black crystals with dimensions up to 5 mm were separated from the liquid after 1 week and dried at room temperature. In order to grow single crystals of CsPbBr_3_, 5 mmol of CsBr (99.9%, Sigma-Aldrich) and 6 mmol of PbBr_2_ were added to 10 mL of DMSO. The mixture was stirred at room temperature for 24 h. The solution was then titrated using methanol (Sigma-Aldrich) until an orange precipitate started to form. Then, a few drops of DMSO were added to dissolve the precipitate, and the solution was filtrated and placed in a small glass vial. This vial was placed in a second, larger glass vial containing methanol. The orange crystals were harvested after 1 week and dried at room temperature.

## Supporting Information Available

Magneto-optical spectroscopy results for MAPbI_3_ and CsPbBr_3_, derivation of the exciton-polaron model in a magnetic field, description of two-band and four-band **kp** model, Comparison of Bajaj model prediction with other exciton-polaron models, and details of *ab initio* band structure calculation. (PDF)
