# Supplementary material for: Polaronic Mass Enhancement and Polaronic Excitons in Metal Halide Perovskites
Source: ACS Energy Lett. 2024 May 13;9(6):2696–702. doi: 10.1021/acsenergylett.4c00905 (PMC11187632; doi:10.1021/acsenergylett.4c00905)
Supplement: Supplementary file 1 — nz4c00905_si_001.pdf [file nz4c00905_si_001.pdf]

Supporting Information: Polaronic Mass Enhancement and Polaronic Excitons in Metal Halide Perovskites

Michal Baranowski,[1,*] Andrzej Nowok,[1,2] Krzysztof Galkowski,[1] Mateusz Dyksik,[1] Alessandro Surrente,[1] Duncan Maude,[2] Marios Zacharias,[3] George Volonakis,[4] Samuel D. Stranks,[5,6] Jacky Even,[3] Miroslaw Maczka,[7] Robin Nicholas,[8] and Paulina Plochocka[1,2,†]

[1]*Department of Experimental Physics, Faculty of Fundamental Problems of Technology, Wroclaw University of Science and Technology, 50-370 Wroclaw, Poland*

[2]*Laboratoire National des Champs Magnétiques Intenses, EMFL, CNRS UPR 3228, Université Grenoble Alpes, Université Toulouse, Université Toulouse 3, INSA-T, 38042 Grenoble and 31400 Toulouse, France*

[3]*Univ Rennes, INSA Rennes, CNRS, Institut FOTON - UMR 6082, F-35000 Rennes, France*

[4]*Univ Rennes, ENSCR, INSA Rennes, CNRS, ISCR - UMR 6226, F-35000 Rennes, France*

[5]*Cavendish Laboratory, University of Cambridge, JJ Thomson Avenue, Cambridge CB3 0HE, UK.*

[6]*Department of Chemical Engineering and Biotechnology, University of Cambridge, Philippa Fawcett Drive, Cambridge CB3 0AS, UK*

[7]*Institute of Low Temperature and Structure Research, Polish Academy of Sciences, ul. Okolna 2, 50-422 Wroclaw, Poland*

[8]*Department of Physics, Clarendon Laboratory, University of Oxford, Parks Road, Oxford, OX1 3PU, UK*

(Dated: May 1, 2024)

MAGNETOOPTICAL CHARACTERIZATION OF $MAPbI_3$ AND $CsPbBr_3$

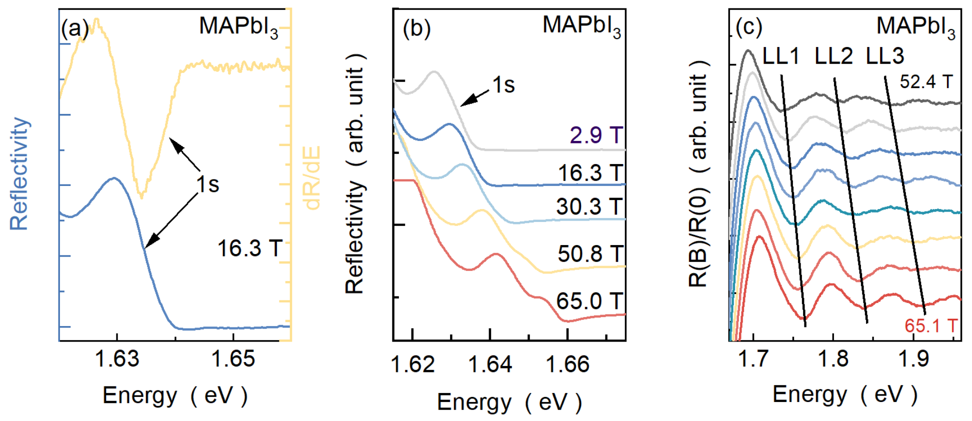

FIG. S1. (a) $MAPbI_3$ reflectance spectrum (blue) taken at 2 K and it's derivative (yellow) showing resonance features related to 1s and 2s excitonic transitions. (b) Evolution of reflectance spectrum in magnetic field. (c) Rationed spectra of reflectance for different magnetic field values, black lines indicated Landau levels (LL) transitions

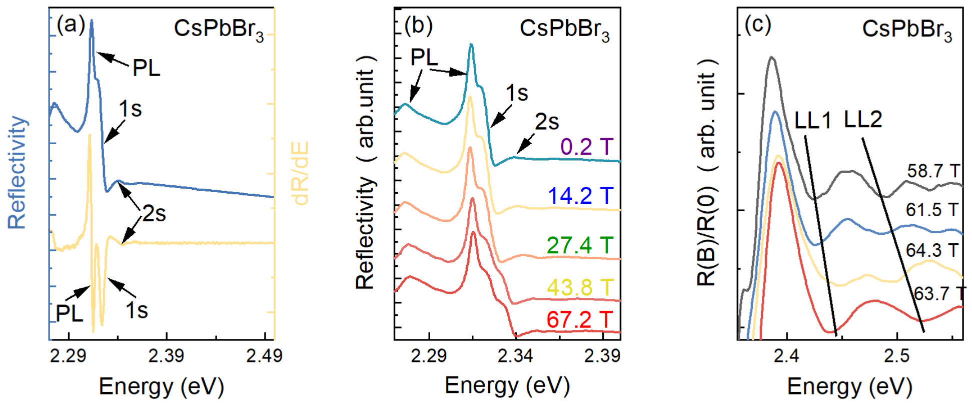

FIG. S2. (a) $CsPbBr_3$ reflectance spectrum (blue) taken at 2 K and it's derivative (yellow) showing resonance features related to 1s and 2s excitonic transitions. (b) Evolution of reflectance spectrum in magnetic field. (c) Rationed spectra of reflectance for different magnetic field values, black lines indicated Landau levels (LL) transitions.

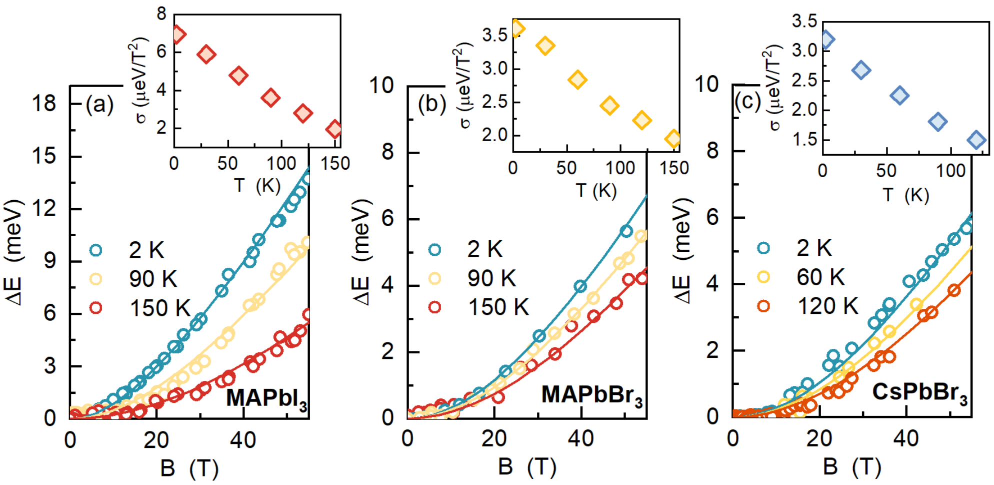

FIG. S3. Excitonic transition shifts measured at different temperatures (open points) for (a) $MAPbI_3$ (b)$MAPbBr_3$ (c) $CsPbBr_3$. In order to quantify the magnetic field-induced shifts the experimental data is fitted assuming for simplicity diamagnetic shift proportional to B^2. The extracted diamagnetic shift coefficients as a function of temperature are presented in the insetes. The decreasing with temperature diamagnetic shifts coefficients σ indicates the rising reduced effective mass of exciton.

EXCITON-POLARON MODEL IN MAGNETIC FIELD

The effective Hamiltonian of exciton-polaron (without magnetic field) proposed by Bajaj[1] has according to form (in a center of mass system):

$$\left(\frac{\mathbf{p}^2}{2\mu^*} + V(R)\right)\Psi(\mathbf{R}) = \left(E - \frac{\hbar^2}{2M}K^2\right)\Psi(\mathbf{R}) \tag{1}$$

where $\mathbf{R} = \mathbf{r_e} - \mathbf{r_h}$ is a relative coordinate of electron and hole, K is a center-of-mass wave vector μ^* is a reduced polaron mass:

$$1/\mu^* = 1/m_e^* + 1/m_h^* \tag{2}$$

where:

$$m_{e/h}^* = m_{e/h}\left(1 + \frac{\alpha}{6}\right) \tag{3}$$

and α is a Frohlich coupling constant:

$$\alpha = \frac{e^2}{\hbar}\left(\frac{1}{\varepsilon_\infty} - \frac{1}{\varepsilon_s}\right)\sqrt{\frac{m_{e/h}}{2E_{LO}}} \tag{4}$$

Potential $V(R)$ is defined acordingly:

$$V(R) = -\frac{1}{\varepsilon_s}\frac{e^2}{R^2} - \left(\frac{\varepsilon_\infty}{\varepsilon_s}\right)^\gamma\left(\frac{1}{\varepsilon_\infty} - \frac{1}{\varepsilon_s}\right)\left(e^{-r/l_e} + e^{-R/l_h}\right)\frac{e^2}{2R} \tag{5}$$

where $l_{e/h}$ is an electron and hole polaron radius defined as:

$$l_{e/h} = \sqrt{\frac{\hbar^2}{2m_{e/h}E_{LO}}} \tag{6}$$

and $\gamma = 3/5$.

In order to include a magnetic field the momentum operator $\mathbf{p}$ has to be modified accordingly[2, 3]:

$$\mathbf{p} \Rightarrow \mathbf{p} + e\mathbf{A} \tag{7}$$

Which results in additional terms in the eq. 1 related to the magnetic field. In the presence of a magnetic field the eq. 1 takes according form:

$$\left(\frac{\mathbf{p}^2}{2\mu^*} + e\left(\frac{1}{m_h^*} - \frac{1}{m_e^*}\right)\mathbf{A}\cdot\mathbf{p} + \frac{e^2}{2\mu^*}\mathbf{A}\cdot\mathbf{A} - \frac{2e\hbar}{M}\mathbf{K}\cdot\mathbf{A} + V(R)\right)\Psi(\mathbf{R}) = \left(E - \frac{\hbar^2}{2M}K^2\right)\Psi(\mathbf{R}) \tag{8}$$

Because photons carry very small momentum optically active exciton states are only those with $\mathbf{K} \approx 0$ therefore for the case of our investigation we can omit expression proportional to K to get:

$$\left(\frac{\mathbf{p}^2}{2\mu^*} + e\left(\frac{1}{m_h} - \frac{1}{m_e}\right)\mathbf{A}\cdot\mathbf{p} + \frac{e^2}{2\mu^*}\mathbf{A}\cdot\mathbf{A} + V(R)\right)\Psi(\mathbf{R}) = E\Psi(\mathbf{R}) \tag{9}$$

Using the Lorentz gauge the vector potential $\mathbf{A}$ can be expressed as:

$$\mathbf{A} = \frac{1}{2}\mathbf{B} \times \mathbf{R} \tag{10}$$

which leads to:

$$\left(\frac{\mathbf{p}^2}{2\mu^*} + \frac{e}{2}\left(\frac{1}{m_h} - \frac{1}{m_e}\right)\mathbf{B} \cdot \mathbf{L} + \frac{e^2}{8\mu^*}|\mathbf{B} \times \mathbf{R}|^2 + V(R)\right)\Psi(\mathbf{R}) = E\Psi(\mathbf{R}) \tag{11}$$

where $\mathbf{L}$ is the angular momentum operator. For the magnetic field along z direction above equation simplifies to:

$$\left(\frac{\mathbf{p}^2}{2\mu^*} + \frac{e}{2}\left(\frac{1}{m_h} - \frac{1}{m_e}\right)BL_z + \frac{e^2 B}{8\mu^*}\left(x^2 + y^2\right) + V(R)\right)\Psi(\mathbf{R}) = E\Psi(\mathbf{R}) \tag{12}$$

We are only interested in optically active s-type exciton states for which angular momentum projection on the z axis is equal to 0. Thus the only factor proportional to the magnetic field which has to be taken into account for optically active transition is $\frac{e^2 B}{8\mu^*}\left(x^2 + y^2\right)$ leading to exciton-polaron hamiltonian presented in the article:

$$\left(\frac{\mathbf{p}^2}{2\mu^*} + V(R) + \frac{e^2 B^2}{8\mu^*}\left(x^2 + y^2\right)\right)\Psi(\mathbf{R}) = E\Psi(\mathbf{R}) \tag{13}$$

The above eigenvalue problem has been solved numerically with the use of the finite elements methods. We have used the Partial Differential Equation Toolbox provided by Matlab software. The problem was solved in 3D geometry. We use Dirichlet boundary conditions with wave function vanishing for large distances (40 Bohr radius of the hydrogen model exciton with dielectric screening corresponding to ε_∞). We use varying element sizes to speed up calculation with mesh becoming more dense approaching $R = 0$ where wave function varies most rapidly.

2 BAND KP MODEL

In a 2-band **kp** model, when we consider only a single conduction and valence band. The energies of the state (close to the bandgap) are described by the equation:

$$E_{e/h}(k) = -\frac{E_g}{2} \pm \frac{1}{2}\sqrt{E_g^2 + \frac{\hbar^2}{m_0^2}p^2k^2} \tag{14}$$

in the above equation, conduction band edge energy is set to 0. p is the interband matrix element, often expressed as a Kane matrix element $P = (\hbar/m_0)p$ where m_0 is a free electron mass. Expanding for small k values the square root function ones obtain:

$$E_e \approx \frac{\hbar^2 p^2}{4m_0^2 E_g}k^2 \tag{15}$$

Defining the effective mass m_e^* of the electron accordingly:

$$E_e(k) = \frac{\hbar^2}{2m_e^*}k^2 \tag{16}$$

We obtain the relation between the effective mass of the electron and the bandgap:

$$\frac{1}{m_e^*} = \frac{p^2}{2m_0^2 E_g} \tag{17}$$

Similarly, for holes we obtain:

$$-\frac{1}{m_h^*} = \frac{p^2}{2m_0^2 E_g} \tag{18}$$

therefore reduced mass of exciton μ is:

$$\frac{1}{\mu} = \frac{1}{m_e^*} + \left|\frac{1}{m_h^*}\right| = \frac{p^2}{m_0^2 E_g} \tag{19}$$

In the 2-band **kp** model the Kane matrix element of $\sim 9\,\text{eV}\text{Å}$ can successfully describe the reduced exciton mass vs bandgap from $MASnI_3$ up to $MA(Cs)PbBr_3$[4].

4 BAND KP MODEL

In the 4 bands **kp** model (assuming cubic phase) the effective masses of hole and electron can be expressed as[5]:

$$-\frac{1}{m_h} = \frac{1}{m_0} - \frac{2p^2}{3m_0^2}\frac{3E_g + \Delta}{E_g(E_g + \Delta)} \tag{20}$$

$$\frac{1}{m_e} = \frac{1}{m_0} + \frac{2p^2}{m_0^2 E_g} \tag{21}$$

where p is the interband matrix element which can be expressed also as a Kane matrix element $P = (\hbar/m_0)p$. For perovskites, it is established that P is around $5 - 7$eVÅ. Fig.S4 shows a comparison of the effective mase rise with the prediction resulting from the above model. We use the value $\Delta = 1.5eV$ and we slightly vary P for each compound to match the modelled value μ at the lowest temperature with experimentally determined reduced effective mass. Evidently, the **kp** model predictions do not follow experimental observation.

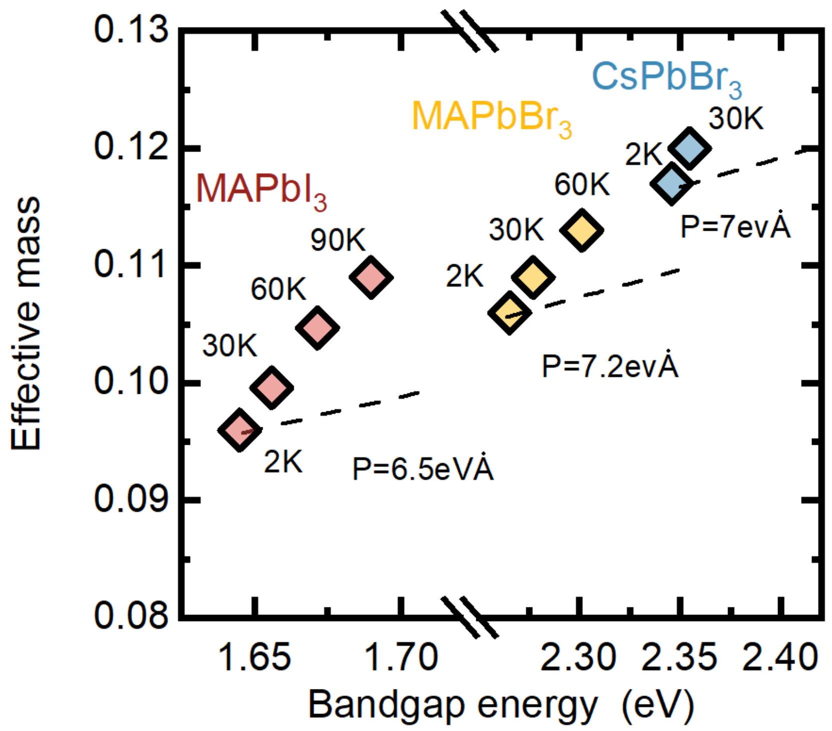

FIG. S4. Comparison of the bare reduced effective mass μ determined experimentally at different temperatures together with the **kp** model prediciton

COMPARISON OF EXCITON BINDING ENERGY FOR DIFFERENT EXCITON POLARON MODELS

To verify how strongly results provided by Bajaj potential[1] deviates from other more sophisticated exciton polaron model we directly compare the result for the same set of parameters as shown in Tab. S1.

	μ	ε_∞	ε_0	E_{LO}	Pollman	Kane	Haken	Bajaj
$MAPbI_3$	0.096	7	15	12	9.7	9.9	15.6	11.3
$MAPbBr_3$	0.106	5.2	16	15	15.8	16.2	32.8	15.2
$CsPbBr_3$	0.117	4.5	16	18	23.3	23.8	52.2	19.4

TABLE S1. Comparison of polaronic exciton binding energies expressed in meV for Pollman[6], Kane[7], Haken[8] and Bajaj[1] models of excion polarons. E_{LO} is optical phonon energy in meV, μ is a reduced mass of carriers (resulting from bare carriers mass). The ε_∞ and ε_0 are optical frequency and static dielectric constant.

BAND STRUCTURE CALCULATION

First-principles calculations of the orthorhombic CsPbBr3 were performed within density functional theory (DFT) using the `Quantum ESPRESSO` code[9, 10]. We employed the Perdew-Burke-Ernzerhof revised for solids (PBEsol) functional[11], optimized norm-conserving Vanderbilt pseudopotentials[12], and a kinetic energy cutoff of 120 Ry. We sampled the Brillouin zone of the unit cell (20 atoms) using a $3 \times 3 \times 3$ uniform **k**-grid. With these settings, the geometry optimization of the orthorhombic $CsPbBr_3$ yields the following lattice constants: $a = 7.97$, $b = 8.40$, and $c = 11.64$ Å. The interatomic force constants were calculated with the frozen phonon method[13] using $2 \times 2 \times 2$ supercells and a $2 \times 2 \times 2$ uniform **k**-grid. The phonon modes and energies at different wavevectors were obtained by Fourier interpolation and diagonalization of the dynamical matrices. To account for long-range dipole-dipole effects, we calculated the Born effective charges and the high-frequency dielectric constant (ε^∞) using density functional perturbation theory[14]. Our calculation yields $\varepsilon^\infty = 4.7$. Fig.S5 shows the phonon dispersion with (black line) and without (red line) corrections due to long-range dipole-dipole interactions. These macroscopic effects lead to

the splitting of the longitudinal optical (LO) and transverse optical (TO) phonons at the Γ point. The LO phonon with the highest energy lies at 18.19 meV. To include the effect of electron-phonon coupling in our electronic structure calculations we employed the special displacement method (SDM)[15, 16] as implemented in the `EPW/ZG` module[17]. Special displacements were generated for a $4 \times 2 \times 2$ supercell (320 atoms) using harmonic phonons carrying the effect of long-range interactions (black lines in Fig. S5). A $1 \times 1 \times 1$ **k**-grid and the effect of spin-orbit coupling (SOC) were used for all SDM supercell calculations. The effective masses were determined by finite differences and taking the average of the diagonal elements of the effective mass tensor.

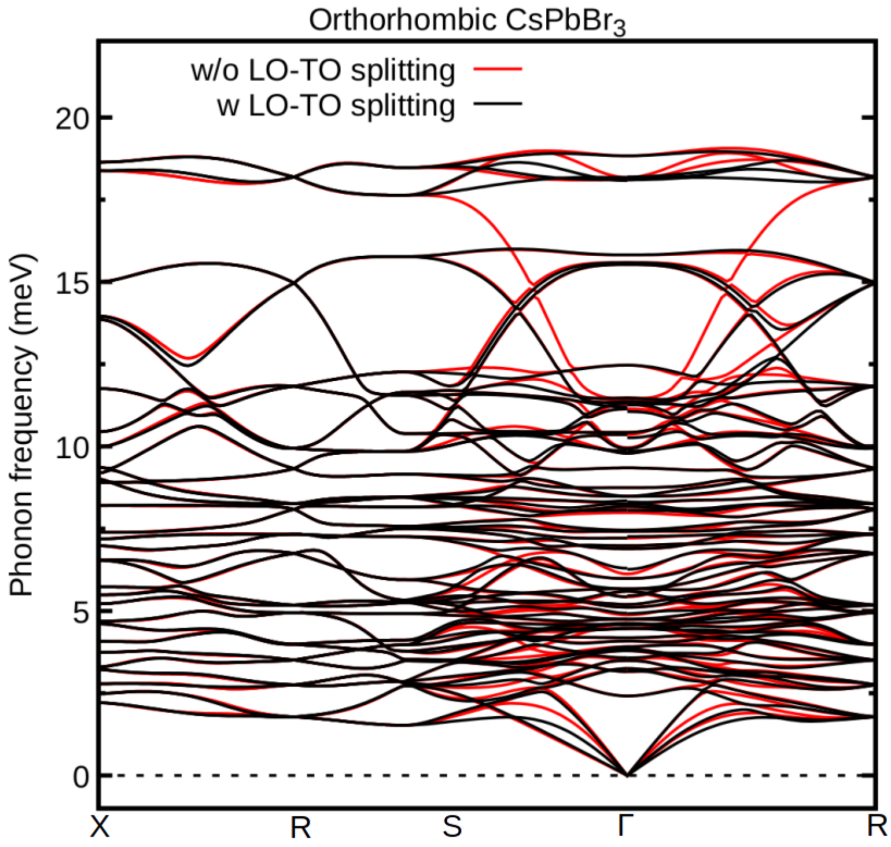

FIG. S5. Phonon dispersion of orthorhombic $CsPbBr_3$ with (black) and without (red) long-range effects.

In Tab.S2 we report band gaps (E_g) of the orthorhombic $CsPbBr_3$ in the temperature range 0–150 K evaluated using the SDM. For completeness, we also report the band gap of the structure at static-equilibrium (E_g^0). Our calculations yield a zero-point renormalization

T	E_g (eV)	$\frac{\Delta E_g}{E_g}$ (%)	m_h	m_e	μ	$\frac{\Delta\mu}{\mu}$ (%)
Equil.	2.331	-	0.185	0.183	0.081	-
0 K	2.326	0.00	0.189	0.187	0.094	0.00
30 K	2.342	0.69	0.196	0.193	0.097	3.16
50 K	2.354	1.19	0.200	0.196	0.099	5.10
75 K	2.369	1.83	0.206	0.202	0.102	8.17
150 K	2.408	3.53	0.227	0.220	0.112	18.46

TABLE S2. Temperature-dependent band gaps (E_g), electron and hole effective masses (m_e and m_h), and reduced effective mass (μ) of orthorhombic $CsPbBr_3$ calculated using the SDM for a $4 \times 2 \times 2$ supercell. Values obtained for the equilibrium geometry, i.e. without including the effect of electron-phonon coupling, are reported. The percentages for the temperature-induced relative increase of the band gap ($\Delta E_g/E_g$) and reduced effective mass ($\Delta\mu/\mu$) are calculated with respect to the 0 K values. All effective masses are in units of the free electron mass (m_0). A PBE0 scissor correction of 1.5 eV is added to our DFT-PBEsol-SOC values[18]

of $E_g(0\,\mathrm{K}) - E_g^0 = -5\,\mathrm{meV}$. We note that our values account for the hybrid functional PBE0 correction of 1.5 eV as calculated in Ref.[18]; this correction yields band gaps of the orthorhombic $CsPbBr_3$ in good agreement with experiment. We also stress that the PBE0 correction to the zero-point band gap renormalization has been found to be negligible[18]. We expect that such a correction to remain nearly the same in the low-temperature regime studied here and not to change quantitatively our calculated temperature-induced relative increase of the band gap ($\Delta E_g/E_g$). In Tab. S2, we also show the electron (m_e) and hole (m_h) effective masses and the reduced effective mass [$\mu = m_e m_h/(m_e + m_h)$]. The calculated values for μ lie within 0.094-0.112 m_0 in the temperature range 0-150 K and underestimate the experimental data. In particular, our calculated μ underestimates measurements both for 2 K and 30 K by 19%. This discrepancy can be adjusted by considering quasiparticle corrections within the GW approximation which have been shown to increase the effective masses by 25%[19]. However, regarding the temperature-induced relative increase of the effective masses ($\Delta\mu/\mu$), quasiparticle corrections are not expected to change the picture, inducing small quantitative changes[20].

In Figs. S6 and S7, we plot data of Tab. S2 to illustrate trends.

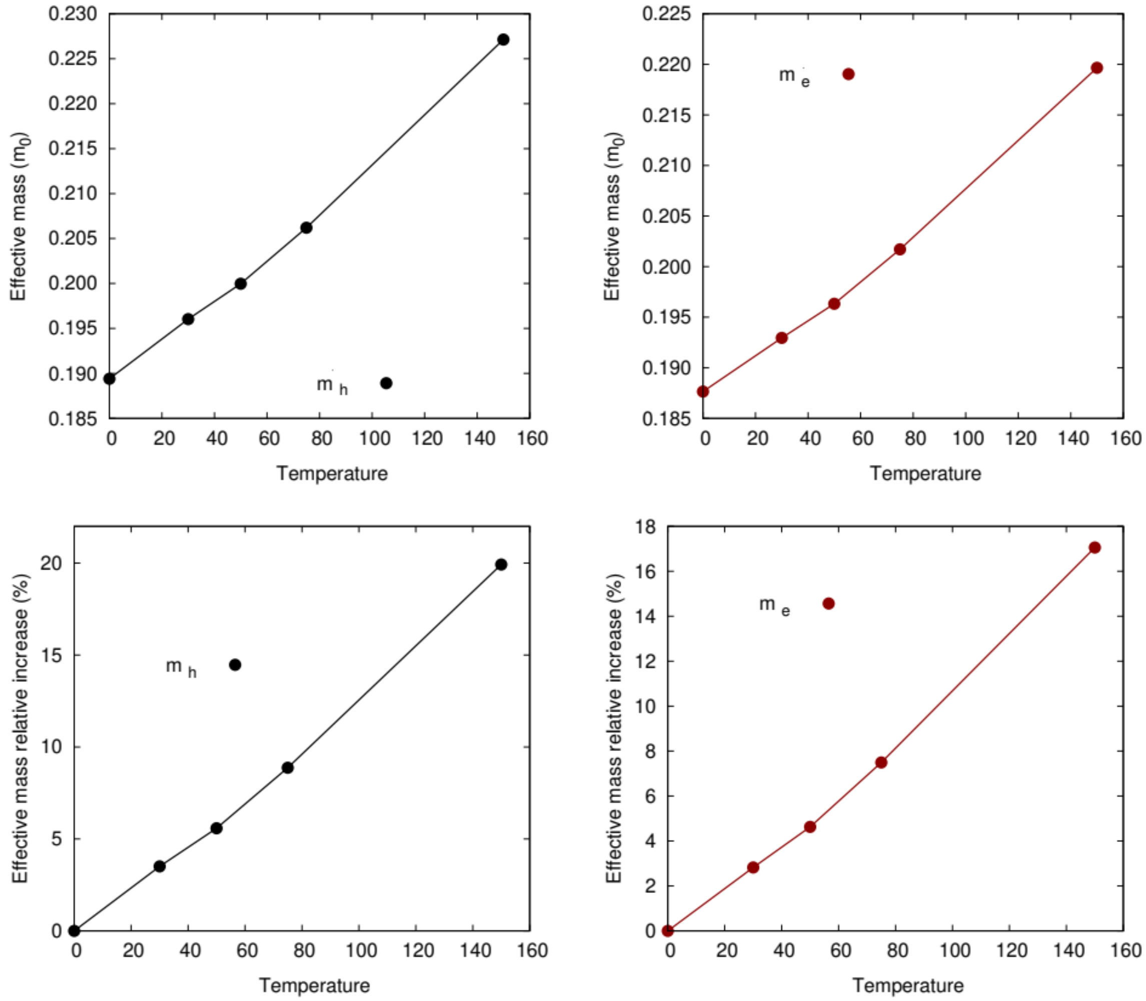

FIG. S6. Electron/hole effective masses (m_e/m_h) of orthorhombic $CsPbBr_3$ as a function of temperature. For each case, we also plot the percentage of the temperature-induced relative increase. Calculations are performed within the SDM framework for a $4\times2\times2$ supercell. All effective masses are in units of the free electron mass (m_0).

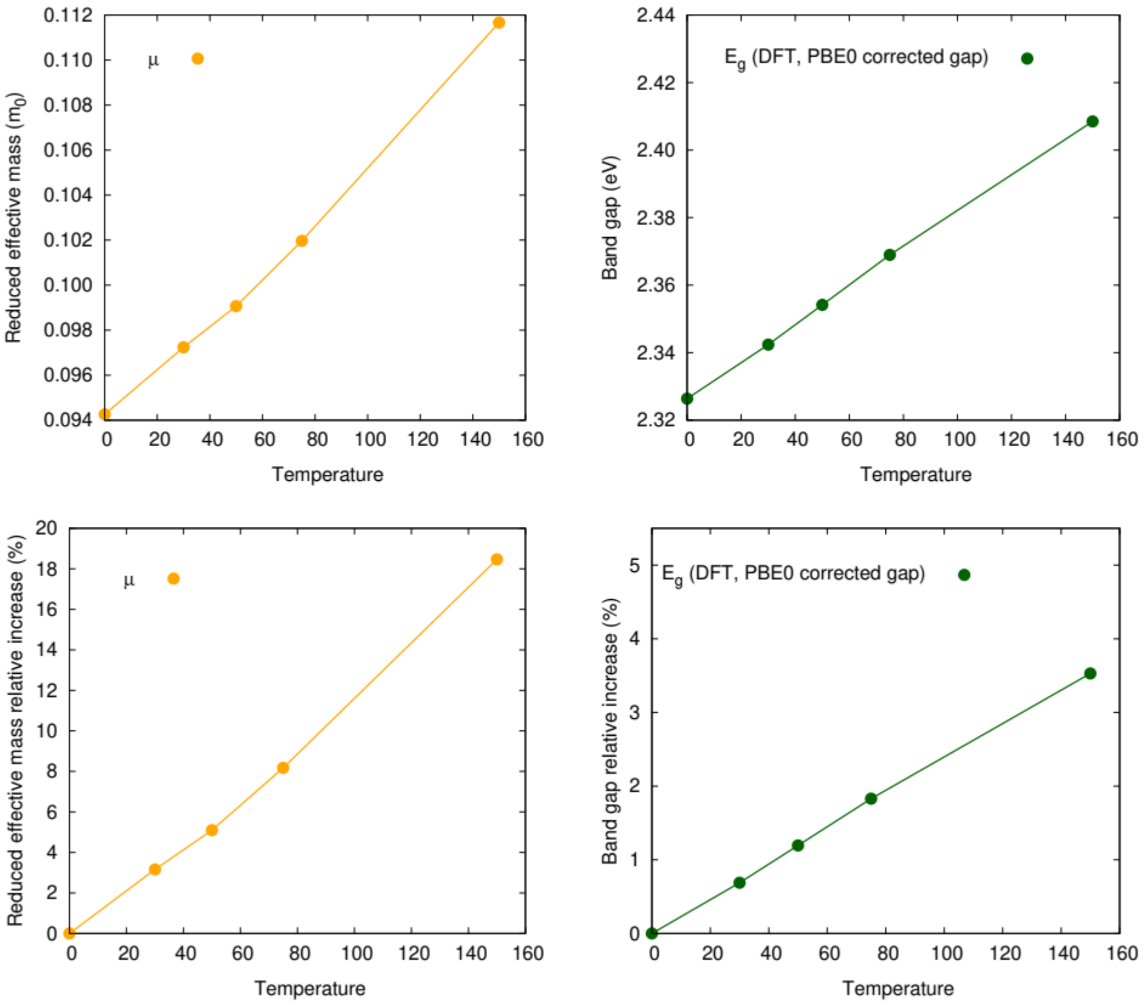

FIG. S7. Reduced effective mass (μ) and band gap (E_g) of orthorhombic $CsPbBr_3$ as a function of temperature. For each case, we also plot the percentage of the temperature-induced relative increase. Calculations are performed within the SDM framework for a $4 \times 2 \times 2$ supercell. Effective masses are in units of the free electron mass (m_0). A PBE0 correction of 1.5 eV is added to our DFT-PBEsol-SOC values[18]

* Corresponding author:michal.baranowski@pwr.edu.pl

† Corresponding author:paulina.plochocka@lncmi.cnrs.fr

[1] K. Bajaj, Effect of electron-phonon interaction on the binding energy of a wannier exciton in a polarizable medium, Solid State Communications **15**, 1221 (1974).

[2] N. Miura, *Physics of semiconductors in high magnetic fields*, Vol. 15 (OUP Oxford, 2007).

[3] J. Singh, *Electronic and optoelectronic properties of semiconductor structures* (Cambridge University Press, 2007).

[4] K. Galkowski, A. Surrente, M. Baranowski, B. Zhao, Z. Yang, A. Sadhanala, S. Mackowski, S. D. Stranks, and P. Plochocka, Excitonic properties of low-band-gap lead–tin halide perovskites, ACS Energy Letters **4**, 615 (2019).

[5] E. Kirstein, D. R. Yakovlev, M. M. Glazov, E. A. Zhukov, D. Kudlacik, I. V. Kalitukha, V. F. Sapega, G. S. Dimitriev, M. A. Semina, M. O. Nestoklon, *et al.*, The landé factors of electrons and holes in lead halide perovskites: universal dependence on the band gap, Nature communications **13**, 3062 (2022).

[6] J. Pollmann and H. Büttner, Effective hamiltonians and bindings energies of wannier excitons in polar semiconductors, Physical Review B **16**, 4480 (1977).

[7] E. O. Kane, Pollmann-büttner variational method for excitonic polarons, Physical Review B **18**, 6849 (1978).

[8] H. Haken, Zur quantentheorie des mehrelektronensystems im schwingenden gitter. i, Zeitschrift für Physik **146**, 527 (1956).

[9] P. Giannozzi, S. Baroni, N. Bonini, M. Calandra, R. Car, C. Cavazzoni, D. Ceresoli, G. L. Chiarotti, M. Cococcioni, I. Dabo, *et al.*, Quantum espresso: a modular and open-source software project for quantum simulations of materials, Journal of physics: Condensed matter **21**, 395502 (2009).

[10] P. Giannozzi, O. Andreussi, T. Brumme, O. Bunau, M. B. Nardelli, M. Calandra, R. Car, C. Cavazzoni, D. Ceresoli, M. Cococcioni, *et al.*, Advanced capabilities for materials modelling with quantum espresso, Journal of physics: Condensed matter **29**, 465901 (2017).

[11] J. P. Perdew, A. Ruzsinszky, G. I. Csonka, O. A. Vydrov, G. E. Scuseria, L. A. Constantin, X. Zhou, and K. Burke, Restoring the density-gradient expansion for exchange in solids and

surfaces, Physical review letters **100**, 136406 (2008).

[12] D. Hamann, Optimized norm-conserving vanderbilt pseudopotentials, Physical Review B **88**, 085117 (2013).

[13] A. Togo and I. Tanaka, First principles phonon calculations in materials science, Scripta Materialia **108**, 1 (2015).

[14] S. Baroni, S. De Gironcoli, A. Dal Corso, and P. Giannozzi, Phonons and related crystal properties from density-functional perturbation theory, Reviews of modern Physics **73**, 515 (2001).

[15] M. Zacharias and F. Giustino, One-shot calculation of temperature-dependent optical spectra and phonon-induced band-gap renormalization, Physical Review B **94**, 075125 (2016).

[16] M. Zacharias and F. Giustino, Theory of the special displacement method for electronic structure calculations at finite temperature, Physical Review Research **2**, 013357 (2020).

[17] H. Lee, S. Poncé, K. Bushick, S. Hajinazar, J. Lafuente-Bartolome, J. Leveillee, C. Lian, J.-M. Lihm, F. Macheda, H. Mori, H. Paudyal, W. H. Sio, *et al.*, Electron–phonon physics from first principles using the epw code, npj Computational Materials **9**, 156 (2023).

[18] M. Zacharias, G. Volonakis, F. Giustino, and J. Even, Anharmonic electron-phonon coupling in ultrasoft and locally disordered perovskites, npj Computational Materials **9**, 153 (2023).

[19] M. Sajedi, M. Krivenkov, D. Marchenko, J. Sánchez-Barriga, A. K. Chandran, A. Varykhalov, E. D. Rienks, I. Aguilera, S. Blügel, and O. Rader, Is there a polaron signature in angle-resolved photoemission of cspbbr 3?, Physical Review Letters **128**, 176405 (2022).

[20] M. Schlipf, S. Poncé, and F. Giustino, Carrier lifetimes and polaronic mass enhancement in the hybrid halide perovskite ch 3 nh 3 pbi 3 from multiphonon fröhlich coupling, Physical Review Letters **121**, 086402 (2018).
